# Diagnostics of Amyotrophic Lateral Sclerosis: Up to Date

**DOI:** 10.3390/diagnostics11020231

**Published:** 2021-02-03

**Authors:** Ivana Štětkářová, Edvard Ehler

**Affiliations:** 1 Department of Neurology, Third Faculty of Medicine, Charles University and Faculty Hospital Královské Vinohrady, 100 34 Prague, Czech Republic; 2 Neurological Department, Faculty of Health Studies, Pardubice University and Pardubice Regional Hospital, 530 03 Pardubice, Czech Republic; edvard.ehler@nempk.cz

**Keywords:** amyotrophic lateral sclerosis, ALS, FTLD, diagnostics, electromyography, biomarkers

## Abstract

Amyotrophic lateral sclerosis (ALS) is a progressive neurodegenerative disease characterized by gradual loss of upper and lower motor neurons and their pathways, usually without affecting the extraocular and sphincter muscles. The cause of the disease is not yet known. It is a chain of subsequent events, ending in programmed cell death in selective neuronal subpopulations. The prognosis for survival is rather short with a median of 2 to 4 years. Survival may be prolonged based on prompt diagnosis, ALS subtype and proper management with supportive treatment (tracheostomy, gastrostomy, etc.). According to the clinical picture, the typical form of ALS with upper and lower motoneuron involvement and progressive bulbar paralysis with bulbar muscle involvement is observed. The ALS form with progressive muscle atrophy, where only the lower motoneuron is affected, and primary lateral sclerosis with only upper motoneuron damage are rare. Familiar forms of ALS (FALS) associated with specific genes (the most common is C9orf72) have been discovered. FALS is usually associated with dementia (frontotemporal lobar dementia, FTLD), behavioral disorders, cognitive dysfunction and impairment of executive functions. The diagnosis of ALS is determined by excluding other conditions and utilizing clinical examinations, laboratory and genetic tests and nerve conduction/needle electromyography studies (EMG). Needle EMG records abnormal activities at rest and looks for neurogenic patterns during muscle contraction. Motor evoked potentials after transcranial magnetic stimulation remain the test of choice to identify impairment of upper motor neurons. New biochemical, neurophysiological and morphological biomarkers are extensively studied as early diagnostic and prognostic factors and have implications for clinical trials, research and drug development.

## 1. Introduction

Amyotrophic lateral sclerosis (ALS) is recognised as a multisystem neurodegenerative disorder, with disease heterogeneity at the clinical, genetic and neuropathological levels [1]. Neurodegenerative disorders are sporadic diseases, some with a hereditary background, with increasing prevalence with age. They develop due to excessive apoptotic death of neurons, which probably occurs due to the accumulation of abnormal protein aggregates in the cytoplasm or nucleus of the cell, but also in the extracellular space [2]. Oxidative stress plays an important role [3]. Mitochondria are the source of cellular energy, but they also produce free reactive oxygen species [4]. Various gene polymorphisms and genome involvement by pathogenic mutations also have an influence, which ultimately facilitates or accelerates the formation of protein aggregates [1,5,6]. The combination of all the above-mentioned pathophysiological mechanisms leads to neuronal damage caused by the loss of functional proteins and thus loss of function. The neuron is also damaged by the toxic effects of newly formed pathological protein aggregates (so-called gain-of-function), especially by accumulation of TDP-43 (transactive response DNA binding protein-43). TDP-43 is localised in the nucleus. It regulates the gene transcription and splicing of mRNA, in addition to the maintenance of mRNA stability [7], which implies that abnormal RNA metabolism is a pivotal event. In about 50% of patients with frontotemporal lobar dementia (FTLD) and more than 90% of patients with ALS, TDP-43 is the most responsible protein forming ubiquitinated inclusions [7,8].

## 2. Definition

ALS is a progressive, paralytic disorder characterised by degeneration and loss of motor neurons in the brain (pyramidal cells in the cortex, corticospinal tract) and spinal motoneurons, usually with the sparing of the extraocular and sphincter muscles [1,5,9].

ALS has been considered predominantly as a disorder of the motor neurons, but recent studies demonstrate the possibility of the non-cell-autonomous pathogenic origin of the disease [10]. Early pathogenic processes involve axonal degeneration, loss of axons and impairment of nerve terminals, anticipating motor neuron impairment, and the onset of clinical signs and symptoms. Dysfunction of motoneuron homeostasis, axonal transport and insufficient energy supply, damage of neuromuscular junction and aberrant axonal branching have been observed in genetic and pathological studies of ALS patients [11].

Sensory impairment can rarely be detected clinically, although subclinical dysfunction of the somatosensory system has already been documented using neurophysiological methods and magnetic resonance diffusion tractography (DTI) [12,13,14]. In neurophysiological studies, functional evaluation of the sensory cortex showed disinhibition in the somatosensory cortex [15,16] that raised the question of whether central somatosensory disinhibition is a primary characteristic of ALS as one element of a multisystem neurodegenerative disorder or a compensatory up-regulation due to functional motor lesions [17]. In human studies, predominant intradermal small fibre loss and axonal sensory neuropathy have been reported [18]. This observation of non-motor system involvement should lead to re-considering ALS as not only a pure motor neuron disease [19].

The revised consensus criteria for the concept of the frontotemporal spectrum disorder of ALS (ALS-FTSD) with the neuropsychological profile including deficits in social cognition and language were presented recently [20]. Evidence shows that the neuropsychological deficits in ALS are extremely heterogeneous, affecting over 50% of persons with ALS, adversely impacting patient survival. Fang et al. [21] performed a systematic review to determine the prevalence of non-motor symptoms in ALS, such as gastrointestinal (sialorrhea, constipation, diarrhoea), autonomic (pain, dyspnoea, urinary incontinence), psychiatric (depression, anxiety, sleep problems, fatigue, suicidal tendency, cognitive–behavioural impairment), vascular (dyslipidemia), itching and pressure sores, which may cause significant distress, worsen prognosis and affect survival. They reported these symptoms in between 5% and 80% of people with ALS. Less recognised and still significant comorbidities that ALS patients may present are prior or concomitant psychiatric illness, such as psychosis and schizophrenia, or mood disorders [22]. A diagnosis of depression was significantly associated with a first record of ALS ≥ 5 years later [23].

In ALS patients without dementia, slight impairment in memory function with abnormal retrieval processes has been documented by SPECT hypoperfusion in the frontal and temporal areas [24].

## 3. Classification

ALS belongs to the “MND” group of diseases (motor neuron disease) [1,5,25]. The phenotypic variability of ALS is wide and includes differences in age and site of onset, the upper and/or lower motor neuron involvement, disease progression and time of survival. Distinct clinical neurological syndromes are characterised by the predominance of upper motor neuron (UMN) degeneration (primary lateral sclerosis, PLS), lower motor neuron (LMN) impairment (progressive muscular atrophy, PMA) or a combination of both UMN and LMN degeneration. The most frequent phenotype, namely, the classic form of ALS, affects the upper and lower motor neurons and occurs in 65–70% of cases. Progressive bulbar paralysis with bulbar muscle involvement occurs in 25% of people. PMA affecting only LMN is rarer with a 5–8% occurrence rate. An extremely rare form is PLS, characterised by the loss of only UMN, which occurs in 1–2% of people. This phenotype displays a relatively long survival [26]. It has been recently published that both ALS and PLS patients exhibited focal thalamus atrophy showing extrapyramidal motor degeneration [27]. Reduced volumes were noted in the amygdala in ALS patients without C9orf72; however, in ALS patients carrying the GGGGCC hexanucleotide repeats in *C9orf72*, abnormalities in thalamic and amygdala nuclei were observed. These data demonstrate genotype-specific patterns in ALS [27].

Variants with focal muscle atrophy, monomelic spinal amyotrophy, brachial amyotrophic diplegia, also known as flail arm, or leg amyotrophic diplegia are very rare; however, an early and prominent involvement of the respiratory muscles is characterised by the worst prognosis [28]. However, mechanical ventilation (tracheostomy-assisted ventilation and non-invasive ventilation) significantly prolongs survival and quality of life in ALS in the subgroup of people with less impaired bulbar function, but not in those with severe bulbar impairment [29].

“ALS plus” syndromes with cognitive impairment form a group of frontotemporal lobar degenerations (FTLD-MND) [5] and share a common factor—the presence of ubiquitin inclusions in the nuclei/cytoplasm of motor neurons, but also in other neurons and in the glia [30,31]. These are the most frequent aggregates of the TDP-43 protein from the group of RNA-/DNA-binding proteins. This protein can be prion-like propagated via the extracellular space by vesicular exocytosis between individual neurons or remotely through the corticospinal pathway [32]. Another pathogenic protein is called FUS (fused-in-sarcoma protein), but its presence is substantially less common compared to TDP-43 [33].

## 4. Prognosis

Individual ALS subtypes have different prognoses. All the above-mentioned subgroups can result in the typical ALS form with UMN and LMN involvement. The disease is fatal, progressive in most patients and death is mostly attributed to respiratory failure. The cause is unknown. The median survival is 2–4 years [6,34]. If ALS patients choose to undergo tracheostomy, they may live, on average, 2 additional years [35].

About 50% of people die within three years of the onset of the first symptoms, 90% die within 5 years and only about 5–10% live more than ten years after the onset of the disease [1,6]. Younger individuals and patients for whom the diagnosis took longer tend to survive longer. The predominance of UMN involvement over LMN damage is also more favourable for long-term survival [36]. Older age and the presence of definite ALS at diagnosis are poor prognostic predictors [37]. Short survival is common in the elderly, who develop bulbar symptoms and respiratory problems early. In ALS-FTLD, patients with an initial motor presentation have a much faster progression than those with a cognitive presentation [38]. These findings suggest that disease progression in ALS-FTLD may be critically linked to physiological and motor changes [39].

The metabolic index seems to be important for prognosis in ALS, when hypermetabolic ALS patients have a greater level of LMN involvement, faster rate of functional decline and shorter survival [40]. Non-invasive ventilation (NIV) and percutaneous gastrostomy are guideline-recommended interventions for ALS symptom management and their use prolongs survival in ALS; however, the risk of bronchopneumonia as the cause of death may be increased by NIV [41]. Frontotemporal syndrome in ALS, defined as behavioural changes or cognitive impairment, is associated with poor survival [42].

## 5. Incidence and Prevalence

In 2015, the estimated prevalence of ALS cases was 5.2 per 100,000 population, affecting people of all races and ethnicities; however, whites, males, non-Hispanics, subjects aged ≥60 years and those with a family history of ALS were more likely to develop the disease [43]. The incidence in Europe is reported to be around 1–2/100,000 inhabitants/year [1], with 2.2 per 100,000 person-years (py) for the general population [44]. In contrast, other population-based studies have measured the lowest incidence in East Asia to be 0.89 per 100,000 py and in South Asia to be 0.79 per 100,000 py [44]. The prevalence in Europe is 4–6/100,000 inhabitants, and the median prevalence is 5.4/100,000 inhabitants [26]. People between the ages of 60 and 70 are usually affected; the disease only rarely develops before the age of 40 [45]. About 5–10% are familial forms, other cases are sporadic [6]. The highest prevalence of ALS is reported from Japan (9.9/100,000 population) with the highest peak at 70–79 years [46]. The lowest incidence is reported in Iran with 0.4/100,000 population [47].

## 6. Aetiology

The cause of this disease has not yet been established. Viral effects, exotoxin effects, including glutamate- and homocysteine-mediated excitotoxicity, failure of proteostasis, mitochondrial dysfunction and oxidative stress, oligodendrocyte dysfunction, cytoskeletal disturbances and axonal transport defects, disturbed RNA metabolism, nucleocytoplasmic transport deficits and impaired DNA repair or immune system dysfunction inducing chronic inflammation have been considered, but these hypotheses have not been validated [1,48,49]. It is a chain of subsequent events, ending in programmed cell death in selective neuronal subpopulations. The sporadic form of ALS (SALS) is the most common one [1,6,43,49].

Other risk factors for ALS development are older age and male sex, body mass index, smoking or blood lipid level [48,50,51]. LDL (low density lipoprotein) is causally associated with ALS and a higher LDL level increases the risk of ALS in both the European and East Asian populations [51]. Physical trauma at younger age may be associated with the development of ALS [52]. Several epidemiological studies have found an association between traumatic brain injury (TBI) and ALS [53], but other studies deny that TBI is an ALS risk [54].

Familial occurrence (FALS) is usually associated with autosomal dominant inheritance with known gene mutations (e.g., superoxide dismutase 1—SOD1, senataxin, dynactin, alsin mutations). These are most often mutations in the C9orf72 [55,56,57], SOD1 and FUS genes [30,31]. ALS is also associated with numerous genes and loci with mutations in DNA/RNA-regulating genes, such as TARDBP [58]. Paraneoplastic aetiology associated with laboratory evidence of well-characterised onconeuronal antibodies anti-Hu, anti-Yo or anti-Ri antibodies is rare, as well as the association of ALS with breast cancer or lymphoma, usually without evidence of onconeural antibodies [59]. The occurrence of selective atrophy of the hypothalamus in both sporadic and familial forms of ALS and in the developed form of FALS in the asymptomatic stage has been recently published [60]. Decreased anterior hypothalamic volume is associated with earlier onset of disease. Noticeable weight loss may precede the onset of the disease by 5–10 years. Hypothalamic atrophy does not correlate with motor impairment. It occurs more in people with early onset.

Early motor manifestations of ALS with the presence of TDP-43 reflect the failure of adaptive complex motor skills. The development of these skills correlates with the development of the motor system unique to primates and significantly improved in humans. Disorders of this system lead to split hand syndrome, gait disorders, split leg syndrome and bulbar signs associated with vocalisation [61].

One of the major pathogenic mechanisms of ALS is mRNA metabolism derangement with miRNA dysregulation due to TDP43 [7,8]. A characteristic feature of TDP-43 protein pathology is its limitation to cortical areas and subcortical nuclei, which are under the direct control of cortical projections. The pathological protein TDP-43 is found in the cerebral cortex, corticofugal fibres and subcortical nuclei and motor neurons of the anterior horns of the spinal cord. The spreading of pathological TDP-43 is assumed by vesicular exocytosis between neurons per continuity and trans-synaptically through corticospinal pathways.

ALS is now considered a primary neurodegenerative disorder involving the concept of prion-like distribution at the synaptic terminals of corticofugal axons [32]. This concept theoretically explains the spread to the neocortex and the association between ALS and frontotemporal dementia.

## 7. Clinical Manifestation

The clinical picture in advanced ALS is rather typical, but the diagnosis may not be clear at the onset of the disease [62]. ALS usually manifests as weakness in the limbs (spinal onset) or difficulty in speaking or swallowing (bulbar onset). Between 58 and 82% of ALS patients have a spinal onset [48]. If bulbar signs with articulation and swallowing problems appear, it is necessary to exclude other disorders of neuromuscular transmission (especially myasthenia), infiltrating tumours and infectious and autoimmune causes (see Table 1) [63].

In patients with predominant UMN involvement, cervical spondylogenic myelopathy and/or radiculopathy, hereditary spastic paraplegia, multiple sclerosis and adrenomyeloneuropathy should be excluded. In subjects with LMN impairment, plexopathy, peripheral neuropathy, myopathies, spinal muscular atrophy, Kennedy’s disease, multifocal motor neuropathy and monomelic amyotrophy should be ruled out.

The disease often begins with an asymmetric weakness in a focal muscle group, most often in the upper limb, with difficultly in writing or unlocking the door, impaired chewing or swallowing, slurred and nasal speech, fasciculations (muscle twitches) in the arm, leg, shoulder or tongue, muscle cramps, stiff muscles, weakness of the dorsal flexion of the hand or plantar flexion of the foot, etc. The clinical finding may resemble mononeuropathy or radiculopathy. Muscle weakness affects neck muscles less often. Marked thoracic kyphosis is noticeable. Muscle fatigue is common. Weight loss is usually caused by muscle atrophy, but also by the disease itself. Fasciculations or cramps appear in the plexus muscles of the upper and lower limbs (deltoid muscle, quadriceps femoris muscle). Atrophies of the small muscles of the hand and foot (especially the interosseous muscles) and generalised fasciculations in the plexus muscles may be found in the clinical picture, leading to substantial discomfort in some patients. Fasciculations may be rarely found in trunk muscles; however, atrophy and fasciculations of the tongue can often be observed in bulbar onset.

ALS with bulbar involvement presents commonly as dysarthria or dysphagia and less often with dysphonia or chewing problems. Axial muscle weakness with posture problems and head drop should be observed in later stages of the disease.

The objective neurological finding in the developed ALS form is characterised by mixed central and peripheral quadriparesis, bulbar signs, quadruhyperreflexia, positive pyramidal signs, atrophy of muscles of the upper and lower limbs and tongue and massive muscle fasciculations, especially on the limbs and in the tongue. There are no sphincter problems and sensory function is usually normal. Executive and cognitive performance is impaired in the ALS form associated with dementia (FTLD-ALS) and behavioural dysfunction develops. The patients are not able to tend to themselves in advanced stages of the disease and become immobile. They are not able to ingest liquid or solid food.

The diagnosis of ALS can be difficult in the early stage of disease, with slow disease progression, or if other neurological diseases are present at the same time. The probability of misdiagnosis, including ALS-mimicking syndromes, is reported to be about 7–8% [62]. Therefore, it is important to rule out these ALS-mimicking syndromes as soon as possible, as delay in treatment may unfavourably affect the outcome [6].

## 8. Diagnostics

Diagnosing ALS is a demanding process in which other disorders with similar clinical manifestations must be reliably ruled out. Reaching a definitive diagnosis and then breaking the news to the patient is a difficult task. For this reason, it is important to send the patient to a high-ranking clinical location with extensive experience diagnosing ALS to get a second opinion as a regular approach that should be implemented [64].

Reaching a diagnosis for ALS is set on three main principles [65]:

(1) Symptoms of functional impairment of a certain area of the body;
(2) Presence of manifestations of central and peripheral motor neuron involvement in one or more segmental anatomical areas;
(3) Progression of functional impairment.

Without the fulfilment of these three requirements, a diagnosis of ALS is considered uncertain and requires new evaluation, although it may not necessarily be erroneous.

### 8.1. Electromyography

Electromyography (EMG) and conduction studies are the basic aids in diagnosis and serve both to identify diseases that mimic ALS and to demonstrate the loss of motor units, which is the basic defining characteristic of the pathogenesis of ALS. The structure of the diagnostics of ALS is based on the revised El Escorial criteria [66]. The anatomical motor area is divided into four regions—bulbar, cervical, thoracic and lumbosacral. In these areas, it is necessary to prove the involvement of UMN and LMN. The involvement of UMN is manifested by spastic paresis, light reflexes with a small amplitude and, following a more detailed examination, a reduced threshold of myotatic reflexes, often in markedly atrophied muscles.

EMG is used to detect lesions of LMN. Conduction study shows normal conduction in sensitive and motor fibres at the onset of the disease. The disease progression with muscle atrophy is associated with the decrease in amplitudes of motor responses and the conduction speed also slows down slightly. This is due to the loss of the fastest motor fibres. A motor neuron lesion may be found through an examination of clinically normal muscles with a needle electrode. With the help of EMG, it is possible to reliably exclude ALS-mimicking conditions—myasthenia, myositis, motor neuropathy and conduction block.

In motor neuron lesions, the distal muscles are very often the first and most affected ones. In these cases, it is necessary to carefully examine the proximal muscles and look for a possible conduction block. It is necessary to perform conduction studies not only for motor fibres but also for sensitive ones. F-waves must also be examined, as a reduced number of F-waves in clinically normal muscles may indicate a conduction block. It should be taken into account that about 20% of patients with ALS have impaired conduction of sensitive fibres [65].

The split-hand index (SI) is another simple neurophysiological measurement that could be utilised in a standard EMG setting. The SI was defined by dividing the amplitude of the compound muscle action potential (CMAP) recorded over the first dorsal interosseous and abductor pollicis brevis by the CMAP amplitude recorded over the abductor digiti minimi. The SI was significantly reduced in ALS patients, especially in those with limb onset [67]. The SI can, in early diagnosis of ALS, differentiate ALS from mimic disorders.

An essential step in using the Awaji-Shima criteria [68] is to examine a sufficient number of muscles with a needle electrode. Fibrillation and sharp waves are typical for active denervation lesions. However, this finding is not typical of proximal muscles, muscles innervated by cranial nerves and muscles that continue to exhibit normal strength and look clinically normal. The finding of fasciculations in these muscles and the evaluation of fasciculations as a manifestation of an active denervation lesion with the simultaneous occurrence of already subacute or chronic neurogenic changes in MUP (higher, often polyphasic, unstable shape, with faster burning > 15 Hz) are the breakthrough points of the Awaji-Shima criteria. Fasciculations therefore have the same diagnostic value as fibrillation or positive waves if neurogenic changes in MUP are present at the same time [65]. The diagnosis of ALS is consequential and must therefore be properly substantiated electrophysiologically, which requires the examination of a significant number of muscles (see Table 2) [69]. Two muscles should always be examined on the limbs, one proximal and one distal. These muscles must not be innervated by the same nerve or the same spinal segment. Evidence of changes in one muscle is sufficient in the area of the cranial nerves and thoracic segments. It is recommended to examine the paravertebral muscles (preferably in the T 6–8 segments) or the straight abdominal muscle.

The cervical and lumbar areas have the highest sensitivity for the detection of peripheral motor neuron disorders. These changes in the cervical and thoracic regions have the highest specificity for ALS [70]. The positive peripherally neurogenic finding varies both according to the type of ALS onset (limb, bulbar) and in relation to the duration of ALS. In asymptomatic limb muscles, EMG changes of 40% have been found [66].

The Awaji criteria allow the diagnosis of ALS to be made earlier and in more patients due to the evaluation of fasciculations. Ultrasound can advantageously be used for the targeted search for fasciculations. Especially in the proximal or larger muscles, the presence of fasciculations is detected by ultrasound. At the same time, it is a non-invasive examination.

The occurrence of fasciculations in patients with ALS is the first sign of motor neuron involvement, reflecting increased axonal excitability. Only later does the instability of MUP appear, and with even longer latency, the development of neurogenic changes in MUP during the extinction of individual motoneurons occurs [71].

Estimation of the number of preserved motor neurons can be conducted using various EMG programs. The original MUNE program (motor unit number estimation) has already been abandoned. Currently, the MUNIX program (motor unit number index) is used, which uses both the area of the compound muscle action potential and the area and amplitudes of the MUP and other characteristics [72].

Revised El Escorial criteria are used for the diagnosis, which divide the ALS forms into clinically definitive, clinically probable, clinically probable ALS laboratory supported (EMG) and possible ALS. Awaji-Shima criteria have been used since 2008; they are more sensitive for patients with bulbar signs [71]. Awaji criteria consider the electrophysiological evidence of the LMN lesion at the same level as the clinical signs. The category of clinically probable ALS laboratory supported was completely removed. The presence of fasciculations was recognised as a manifestation of the LMN lesion. These criteria enable the much earlier diagnosis of ALS in many patients, and there are not as many false-positive diagnoses of ALS [73].

### 8.2. Transcranial Magnetic Stimulation

Motor evoked potentials (MEP) after transcranial magnetic stimulation (TMS) confirm the lesion of the UMN or corticospinal pathway. ALS is characterised by cortical hyperexcitability with an increased motor threshold and dysfunction of intracortical inhibition (especially short interval intracortical inhibition—SICI) after TMS [74]. It has been recently published that short- and long-latency afferent inhibitions after TMS were both impaired in ALS, probably unrelated to increased cortical excitability or cognitive dysfunction [75]. Dysfunction of transcallosal circuits using TMS has been observed as an important pathophysiological mechanism in ALS, correlating with greater disability and a faster rate of disease progression [76].

Threshold tracking methodologies have been recently adopted for TMS, whereby changes in threshold rather than MEP amplitude serve as outcome measures [77,78]. Cortical hyperexcitability as an important pathogenic mechanism in ALS was demonstrated as an early feature in sporadic ALS preceding the onset of LMN dysfunction and correlating with neurodegeneration and disease spread [74].

### 8.3. Biomarkers

These days, determination of suitable biomarkers in ALS is an important issue for practical management of ALS, providing significant potential for diagnostics, prediction of disease course and optimisation of the therapeutic responses.

Advanced magnetic resonance imaging (MRI) methods detect an early degeneration of upper motor neurons as well as other systems involvement such as the sensory system or basal ganglia, demonstrating that ALS is a multisystem disorder [79]. Application of non-conventional magnetic resonance imaging (MRI) such as diffusion tensor imaging (DTI), magnetic resonance spectroscopy (MRS) and magnetisation transfer imaging (MTI) may help to determine the pathophysiological process of ALS [80]. These new imaging techniques have recently been used as non-invasive neurophysiological ALS biomarkers in the stage of diagnosis and monitoring of disease progression [81]. For example, the affection of the corticospinal or corticobulbar pathway can be demonstrated by reduced fractional anisotropy in DTI [82]. Various sequences for magnetic resonance imaging (MRI) of the brain and spinal cord may be used as surrogate biomarkers also in clinical trials [83].

Cerebrospinal fluid (CSF) assessment is the most important at the early stages of the disease to exclude other neurological disorders mimicking ALS. CSF neurofilaments (NF), TDP-43 and the tau protein serve as diagnostics biomarkers [84] and may be used as valuable markers of disease progression. For example, the relation between the high levels of CSF tau and short survival in ALS has been reported [85]. Neurofilament light chain (NfL) and phosphorylated neurofilament heavy chain (pNfH) in CSF and serum have been considered as possible diagnostic and prognostic biomarkers for ALS [86].

Over the last decade, changes in three major lipid species, namely, cholesterol, triglycerides and fatty acids, have been proposed as other potential biomarkers of ALS [87].

Neuroinflammation may play an important role in the pathogenesis of ALS. This contribution is supported by findings of alterations in levels of numerous inflammatory cytokines; however, none of them are sensitive and specific enough to become a universal biomarker for ALS [88].

Some authors have been investigating the levels of muscle-specific microRNAs (myoMiRnas) in the serum of ALS patients and found that muscle mass regulators are particularly down-expressed in bulbar ALS, suggesting a more rapid and diffuse atrophic process [89]. Differences in myomiRNAs were found in ALS muscles according to gender, age at onset and disease duration and between familial and sporadic forms [90,91].

Neurophysiological biomarkers of LMN dysfunction, including motor unit number estimation, the neurophysiological index, electrical impedance EMG and axonal excitability techniques, can be easily used to monitor the progression of ALS [78]. Cortical hyperexcitability has been shown to be a suitable diagnostic biomarker of UMN dysfunction, helping to differentiate from neuromuscular mimicking disorders at early ALS stages.

### 8.4. Management

ALS as a progressive neurodegenerative disorder has a substantial impact on quality of life (QoL), directly related to physical integrity and functional independence. Several studies focused on aspects of QoL and independence including respiratory care, mental health, communication skills and exercises [92,93,94].

The most appropriate measures of QoL are the SF-36, a generic widely used QoL measure, and ALSAQ-40, the ALS-specific measure with a forty-item ALS assessment questionnaire [95,96]. Many validation studies on the ALSAQ-40 were undertaken [97,98,99].

There is currently no specific cure for this disease. Supportive and symptomatic treatments provided by a specialist multidisciplinary team are strongly recommended to manage the accompanying symptoms improving survival [100].

## 9. Conclusions

The cause of ALS is not yet known and, unfortunately, there is currently no specific cure for this devastating disease. At the beginning, ALS is still difficult to diagnose. ALS has a short survival and poor prognosis for most patients. New biochemical, neurophysiological and morphological biomarkers are important as early diagnostic and prognostic factors. It is important to create ALS registries with voluntary enrolment of ALS patients for a better understanding of this disease including specific care and therapies. ALS patients should be referred to a multidisciplinary team who will assist them, their families and caregivers with managing the disease.

## Figures and Tables

**Table 1:** Differential diagnosis of ALS [63].

Disease	Differences Compared to ALS
Multifocal motor neuropathy	There is no involvement of bulbar muscles, conduction studies—conduction blocks
Spinal muscular atrophy	Only affects LMN, age-related incidence, SMN detection (genetic testing)
Primary lateral sclerosis	Only affects UMN, slower course, survival over 10 years, MEP—no cortical response
Spinal and bulbar muscular atrophy	It develops in middle age, men, fasciculation of the tongue and in the perioral region, gynaecomastia, X chromosome, expansion in the androgen receptor gene
Hereditary spastic paraplegia	Lower limb spasticity, gait with pelvic rotation, minimal upper limb symptoms, familial occurrence, genetic confirmation
Myogenic lesions (PM, IBM)	Myopathic syndrome, proximal weakness, laboratory findings (CK), muscle MRI, muscle biopsy
Myasthenia gravis	Fatigue, localisation of impairment (ocular, bulbar, head posture), repetitive stimulation, antibodies
Spondylogenic cervical myelopathy	Symptoms—including sensory disorders, sphincter disorders, no bulbar symptoms, MRI finding
Lumbar spinal stenosis	Symptoms of lower extremities only, including sensory or sphincter disorders, fatigue—claudications, MRI findings
Other forms of ALS-like (inflammatory, radiology-induced, paraneoplastic)	Demonstration of the underlying process (tumour, radiology-induced, postpolio syndrome, retrovirus), not continually progressive course, rarely fasciculations; possible sensory neuropathy in the paraneoplastic form, which is very rare

Abreviations: PM—polymyositis, IBM—inclusion body myositis, SMN—spinal motoneuron, CK—creatinkinase, UMN—upper motoneuron, LMN—lower motoneuron, MRI—magnetic resonance imaging, MEP—motor evoked potentials.

**Table 2:** Recommended protocol for EMG examination in suspected ALS/MND according to Awaji-Shima [69].

Region	Muscles
Upper limb, lower limb	Demonstration of changes in one proximal and one distal muscle innervated by different peripheral nerves and from another spinal cord segment
Thoracic area	Changes in one muscle are sufficient. Paraspinal muscles (T5–6) or rectus abdominis is suitable. T11–12 segments are not recommended
Bulbar region	Evidence of changes in one muscle (tongue, masseter, sternocleidomastoid muscle, mimic muscles) is sufficient
